# Tension free open inguinal hernia repair using an innovative self gripping semi-resorbable mesh

**DOI:** 10.4103/0972-9941.27726

**Published:** 2006-09

**Authors:** Philippe Chastan

**Affiliations:** Clinique des 4 pavillons, Rue Edouard Herriot, 33310 Lormont, France

**Keywords:** Inguinal hernia, self-gripping mesh, tension-free repair

## Abstract

**Aims:**

Inguinal hernia repair according to Lichtenstein technique has become the most common procedure performed by general surgeons. Heavy weight polypropylene meshes have been reported to stimulate inflammatory reaction responsible for mesh shrinkage when scar tissue evolved. Additionally, some concerns remain regarding the relationship between chronic pain and mesh fixation technique. In order to reduce those drawbacks, we have developed a new mesh for anterior tension free inguinal hernia repair which exhibits self-gripping absorbable properties.

**Materials and Methods:**

52 patients (69 hernias) were prospectivly operated with this mesh (SOFRADIM-France) made of low-weight isoelastic large pores knitted fabric which incorporated resorbable micro hooks that provides self gripping properties to the mesh during the first months post-implantation. The fixation of the mesh onto the tissues is significantly facilitated. The mesh is secured around the cord with a self gripping flap. After complete tissular ingrowth and resorption of the PLA hooks, the low-weight (40 g/m^2^) polypropylene mesh insures the long term wall reinforcement.

**Results:**

Peroperativly, no complication was reported, the mesh was easy to handle and to fix. Discharge was obtained at Day 1. No perioperative complication occurred, return to daily activities was obtained at Day 5.5. At one month, no neurological pain or other complications were described.

**Conclusions:**

Based on the first results of this clinical study, this unique concept of low density self gripping mesh should allows an efficient treatment of inguinal hernia. It should reduce postoperative complications and the extent of required suture fixation, making the procedure more reproducible

Inguinal hernia repair using Lichtenstein technique has become the most frequently tension free procedure performed by general surgeons. This technique is easy to learn, easy to perform under local anaesthesia and demonstrates a low rate of recurrence.

The most commonly used material in this technique is polypropylene, although published results on multifilament polyester meshes demonstrated safe and efficient results.[1] Heavy weight polypropylene meshes have been reported to stimulate inflammatory reaction responsible for mesh shrinkage when scar tissue evolves.[2] Additionally, some concerns remain regarding the relationship between chronic pain and mesh fixation technique.

In order to reduce those drawbacks some authors have recommended the use of low-weight meshes[3,4] and to limit the extent of fixation or to use non compressive absorbable devices.[5]

In order to reduce these complications and to combine both approaches, we have developed a new mesh for anterior tension free inguinal hernia repair which exhibits self-gripping resorbable properties. The aim of this study is to demonstrate the performance, the tolerance of this innovative mesh and the comfort improvement of the patient.

## MATERIALS AND METHODS

Patients were consecutively included in the protocol and the only exclusion criterion was indication for laparoscopic inguinal hernia repair.

### Description of the mesh

**Figure 1:**
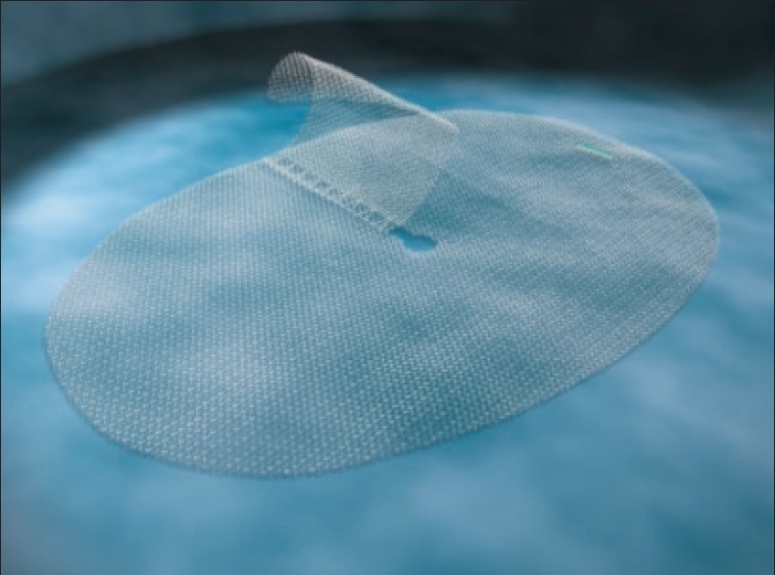
PP 1208 D mesh (Polypropylene + PLA)

**Figure 2:**
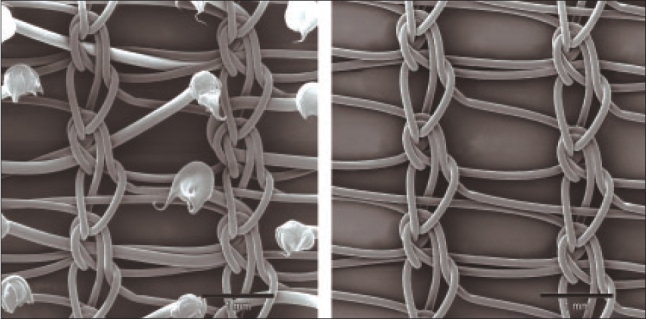
Mesh before and after PLA resorption

The procedures were performed using the new PP1208D proprietary mesh manufactured by SOFRADIM®, France [Figure 1]. The PP1208D mesh is made of a low-weight monofilament polypropylene (PP) knitted fabric which incorporates resorbable polylactic acid (PLA) micro hooks. The polypropylene fabric consists in a low weight isoelastic structure with large pores [Table 1]. The resorbable polylactic acid micro-hooks incorporated in the polypropylene structure provides self gripping properties to the mesh during the procedure and the first month post-implantation [Table 1]. The fixation of the mesh onto the muscle and surrounding tissues is significantly facilitated. The mesh can be secured around the spermatic cord with a self gripping flap that can be easily repositioned. The self gripping flap is made of the same fabric as the mesh, i.e., the polypropylene with polylactic acid micro-hooks knitted fabric. After complete tissue ingrowth and complete resorption of the polylactic acid hooks, the low-weight (40 g/m^2^) polypropylene mesh ensures the long term wall reinforcement [Figure 2].

**Table 1:** Mechanical characteristics and gripping strength evaluation

Mechanical characteristics		
	**New self-gripping mesh**	
Surface density (g/m^2^)	Before PLA resorption	82
	After PLA resorption	41
Thickness (mm)	0.5
Porosity (%)	91
**Gripping strength evaluation**	
	**Gripping strength (N)**
New self-gripping mesh	21.4 ± 5.7
Standard textile	5.2 ± 0.9

### Surgical technique

The surgical technique recommended for this mesh as follows: after a 5 to 8 cm skin incision, the external oblique aponeurosis is incised. The spermatic cord is carefully dissected and the cremasteric muscle resected. The inguinal ligament is dissected towards the pubis up to the anterior superior iliac spine. A wide dissection of the conjoint tendon and the rectus muscle aponeurosis is performed up to the linea alba as to create the space required to spread out the mesh. The pubic bone is dissected and bared about 2 cm. The direct or oblique external sac is never opened; it is replaced in its course.

**Figure 3:**
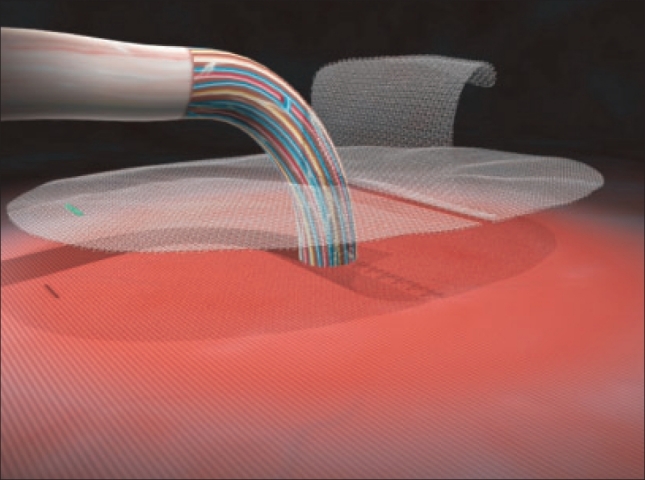
Self gripping mesh: flap opened

**Figure 4:**
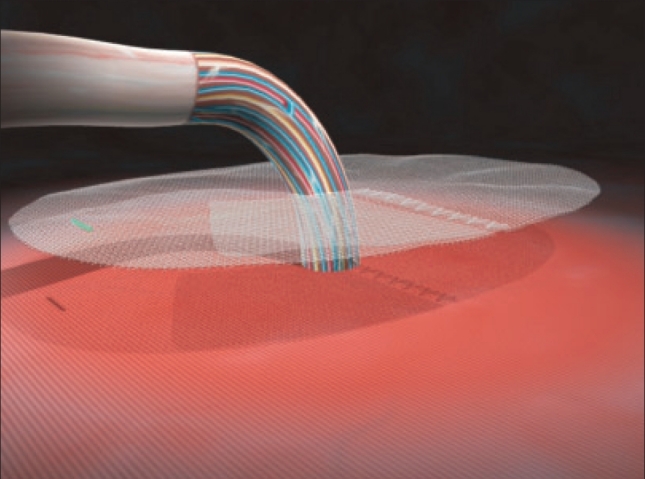
Self gripping mesh: flap closed around the cord

The mesh is then opened from its packing and any folding of the mesh should be avoided. The self gripping flap of the mesh is then opened [Figure 3] and closed around the cord outside the operating area in order to avoid any untimely side by side placement [Figure 4]. The mesh is then spread down carefully to its final position (color stitch orientated toward the pubic bone), its fixation starting inferiorly to the high right muscle and to the adjacent inguinal ligament. In case of large mesh overlap on the inguinal ligament, it should be folded during closure thus allowing minimizing the eventual mesh shrinkage. Thanks to the micro hooks grip, mesh fixation is immediate, no additional fixation suture is required except when the mesh overlap is not sufficient. In this case, one stitch of absorbable suture on the pubic spine may prevent early migration of the mesh. The external oblique aponeurosis is closed retro-funicular by absorbable sutures in order to protect the fragile structures from a direct contact with the mesh.

### Patient data

Fiftytwo patients were included in the study. Data collection used standardized clinical report forms. Preoperative information of the patient was obtained: recurrence and previous treatment, preoperative pain measured via Visual Analogic Scale (VAS) graduated from 0 to 10. Peroperative data was collected: type of hernia, collar diameter, operative time, peroperative complications and surgeon's handling evaluation. Clinical follow up was performed at one month; complications, pain and recurrence were carefully collected.

## RESULTS

The results reported in this paper are based on a one month follow up. Fifty-two male patients representing 69 inguinal hernias were enrolled in the study between February 2006 and June 2006 (mean age was 60 ± 14 years old). Mean body mass index was 24.4 ± 3. 29 (55.8%) of the patients had no professional activity, 5 (9.6%) were independent professionals or were executives, 4 (7.7%) were state employees, 2 (3.8%) were employees, 12 (23.1%) were manual workers. Peroperative pain describe by the patient was: 2.3 ± 2.2.

Local anesthesia was performed in 44% of cases, locoregional anesthesia in 25% and general anesthesia in 31%. 66 patients (95.6 %) were operated for primary hernias and 3 (4.4%) for recurrences. The 3 recurrences were previously treated by Shouldice in two patients and by Bassini lasting one. Description of hernias operated is described in table 2. The mean collar diameter of the hernia was 2.5 cm ± 1.3, no case of strangulated hernia was operated. Mean operating time was 19 ± 4 min. The subjective mesh evaluation by the surgeon is described in Table 3.

**Table 2:** Type of hernia (Nyhus)

Type of hernia	Number of hernias	%
Type 1	3	4.3
Type II	31	45
Type IIIa	12	17.4
Type IIIb	22	31.9
Type IVd	1	3.1

**Table 3:** Subjective mesh evaluation by the surgeon

	Very good	Good	Very bad	Bad
Fixation preciseness	48 (92.3)	3 (5.8)	0	1 (1.9)
Quality of fixation (grip)	51 (98.1)	1 (1.9)	0	0
Manipulability/comfort use	45 (86.5)	7 (13.5)	0	0

Figures indicates in parentheses are percentage

Perioperative results [Table 4]: no perioperative complication was reported, outpatients were 12 (23%), hospital stay was 1 day for 38 patients (73%), 2 (4%) patients were hospitalized for 2 days for personal convenience. The day of discharge 23 patients (44.2%) had taken minor analgesic (Paracetamol) medication. Return to daily activity was effective at day 5.5 ± 3.6 and return to work was obtained at day 20 ± 11.

**Table 4:** Preliminary results

Preliminary results		
**Discharge:**		
Hospitalization	Mean 1 day	
Mean pain (VAS/10)	Left: 1.1 ± 1.2	Right: 1.4 ± 1.4
Early complication		
Cord induration	0	
Hematoma-seroma	0	
Recovery (days)		
Return to normal daily activities	5.5 ±3. 6	
Return at work	20 ± 11	
At one month:		
Mean pain (VAS/10)	Left: 0.3 ± 0.6	Right: 0.1 ±0.4
Hematoma-seroma	0	
Cutaneous infection	1 (cured at 30 days)		
Cutaneaous inflammation	0	
Testicular pain	0	
Induration	2	
Recurrence	0	

VAS - Visual analogic scale

At one month [Table 4]: Only 3 patients (5.7%) took minor analgesics (paracetamol). One cutaneous infection was reported that was cured at day 30, no testicular pain nor recurrence was observed, only 2 indurations were reported.

## DISCUSSION

The preliminary results of this study are promising and show the technical advantages of this innovative self gripping mesh. Operating time is short - 19 min are necessary to perform the surgery (skin incision to closure). The time necessary to spread out the mesh and fix it is less than 1 min with the described technique. Mesh fixation was performed by one stitch only at the beginning of inclusion period for safety reasons. This short time necessary for mesh fixation reduced the time of mesh exposure and could reduce sepsis complications.

No preoperative difficulty or complication was reported. The pain at discharge was low and reduced in comparison with preoperative pain described by patients. The lack of tension during mesh positioning and closure of the flap around the cord can reduce the pain generated by tension created on surrounding tissues and more particularly if sutures can be avoided.

The grip fixation provides the advantage of obtaining fixation on the whole surface of the mesh that can reduce the risk of hernial bag sliding between the device and the transversalis fascia. At one month, no patient reported neurological pain. The fascia protecting the ilio-inguinal nerve can't be penetrated by the hooks.

Chronic pain is perhaps the most serious adverse outcome after inguinal hernia repair, it is reported during daily activities in 16.6% of cases.[6]

With this mesh, we expect to improve quality of life of the patients by reducing post-operative pain. The lightweight mesh can contribute to reduce this adverse outcome,[7] in case of neurological pain, the resorption of fixating grip in about one year should allows a reduction and disappearance of the pain.

## CONCLUSION

This unique concept of low density self gripping mesh should allow an efficient treatment of inguinal hernia treatment via Lichtenstein technique. It should reduce postoperative complications by creating less fibrosis reaction and reducing the extent of required suture fixation. Finally, it should simplify the procedure by making it more reproducible.
